# Differential Effect of Viral Hepatitis Infection on Mortality among Korean Maintenance Dialysis Patients: A Prospective Multicenter Cohort Study

**DOI:** 10.1371/journal.pone.0135476

**Published:** 2015-08-11

**Authors:** Eugene Kwon, Jang-Hee Cho, Hye Min Jang, Yon Su Kim, Shin-Wook Kang, Chul Woo Yang, Nam-Ho Kim, Hyun-Ji Kim, Jeung-Min Park, Ji-Eun Lee, Hee-Yeon Jung, Ji-Young Choi, Sun-Hee Park, Chan-Duck Kim, Yong-Lim Kim, Clinical Research Center for End Stage Renal Disease (CRC for ESRD) Investigators

**Affiliations:** 1 Department of Internal Medicine, Kyungpook National University School of Medicine, Daegu, Korea; 2 Department of Statistics, Kyungpook National University, Daegu, Korea; 3 Department of Internal Medicine, Seoul National University College of Medicine, Seoul, Korea; 4 Department of Internal Medicine, Yonsei University College of Medicine, Seoul, Korea; 5 Department of Internal Medicine, The Catholic University of Korea College of Medicine, Seoul, Korea; 6 Department of Internal Medicine, Chonnam National University Medical School, Gwangju, Korea; 7 Clinical Research Center for End Stage Renal Disease in Korea; 8 BK21Plus KNU Biomedical Convergence Program, Department of Biomedical Science, Kyungpook National University, Daegu, Korea; University of Pisa, ITALY

## Abstract

The role of infection with hepatitis B virus (HBV) and hepatitis C virus (HCV) in terms of survival among dialysis patients remains incompletely understood. In the present multicenter prospective cohort study, we investigated the prevalences of HBV and HCV infection among 3,321 patients receiving maintenance dialysis in Korea, and assessed the impacts of these infections on survival. All included patients underwent hepatitis B antigen (HBsAg) and HCV antibody (Ab) testing, which revealed that 236 patients (7.1%) were HBsAg-positive, and 123 patients (3.7%) were HCV Ab-positive. HBsAg-positive and HCV Ab-positive patients were matched to hepatitis virus-negative patients using a propensity score at a ratio of 1:2. The prevalences of HBV and HCV infection did not significantly differ according to dialysis modality. Linear-by-linear association analysis revealed that hepatitis B prevalence significantly increased with increasing dialysis vintage (*p* = 0.001), and hepatitis C prevalence tended to be higher with increasing dialysis vintage (*p* = 0.074). We compared the survival of HBsAg-positive and HCV Ab-positive patients to that of hepatitis virus-negative patients. After propensity score matching, cumulative survival did not differ between HBsAg-positive and HBsAg-negative patients (*p* = 0.37), while HCV Ab-positive patients showed significantly lower survival than HCV Ab-negative patients (*p* = 0.03). The main conclusions of the present study are that HBV infection prevalence increased with longer dialysis vintage, and that both HBV and HCV infections were most prevalent among patients with the longest dialysis vintage. Additionally, HCV infection among maintenance dialysis patients is associated with an increased risk of mortality.

## Introduction

In many regions of the world, hepatitis B virus (HBV) and hepatitis C virus (HCV) are endemic and represent common causes of chronic hepatitis, cirrhosis, and hepatocellular carcinoma [1]. Korea shows high HBV endemicity, with a hepatitis B surface antigen (HBsAg) seropositivity rate of ≥8% [2, 3]. Among Koreans older than 40 years of age, HCV infection prevalence is estimated to be 1.3% [4]. Both HBV and HCV can be transmitted via infected blood products; thus, patients on maintenance dialysis have a particularly high risk of infection. Among patients receiving maintenance dialysis in Asian-Pacific countries, HBV prevalence ranges from 1.3–14.6%, and the prevalence of HCV antibody (Ab) positivity is between 0.7% and 18.1% [5].

Several studies have investigated how HCV infection influences survival among dialysis patients [6–11]. One meta-analysis shows an association between HCV Ab positivity and increased mortality risk among patients receiving regular dialysis [12]. However, most studies have enrolled Caucasian patients; thus, little information is available regarding Asian patients undergoing maintenance dialysis. Moreover, relative to HCV infection, less is understood about the role of HBV infection in survival among dialysis patients. A study from the United States reports similar death rates between HBsAg-positive and HBsAg-negative long-term dialysis patients [13], while a retrospective study in India reported a higher mortality rate among hepatitis B patients on maintenance dialysis [14]. Since these results were reported, treatment strategies and anti-viral agents have changed. Furthermore, no previous studies have compared the impacts of both HBV and HCV infection on survival in dialysis patients.

The present study aimed to compare the impacts of HBV and HCV infection on survival among patients receiving maintenance dialysis. We enrolled patients with HBV or HCV infection, and used propensity score matching analysis to select patients without viral hepatitis from a multicenter prospective cohort of Korean patients on maintenance dialysis.

## Materials and Methods

### Study Cohort

We conducted a nationwide prospective observational cohort study in Korean patients with end-stage renal disease (ESRD) (NCT00937970). From September 2008 to December 2012, we screened a total of 3,336 patients from 31 centers affiliated with the Clinical Research Center for ESRD. Patients were eligible if they were at least 20 years old, they had started ESRD treatment with maintenance dialysis within 3 months, and they were not scheduled to receive kidney transplantation within 3 months. Fifteen patients with both HBsAg-positive and HCV Ab-positive serology were included only in prevalence analysis. All patients provided written informed consent before inclusion, and the study protocol was approved by the Institutional Review Board of Kyungpook National University Hospital (2011-01-041). All clinical investigations were conducted in accordance with the guidelines of the 2008 Declaration of Helsinki.

### Data Collection

At the time of enrollment, we collected baseline information, including patient age, sex, dialysis modality, comorbidities at dialysis initiation, and laboratory data. Comorbid conditions included history of diabetes, congestive heart failure, coronary artery disease, peripheral vascular disease, arrhythmia, cerebrovascular disease, chronic lung disease, peptic ulcer disease, moderate-to-severe chronic liver disease, and malignancy. Moderate-to-severe liver disease was defined as chronic hepatitis with elevated liver function test results, symptomatic chronic active hepatitis requiring medication, esophageal varices, ascites, liver cirrhosis, history of portocaval shunts, or previous surgical procedure for portal hypertension. Laboratory data included levels of hemoglobin, serum blood urea nitrogen, creatinine, albumin, calcium, phosphorus, aspartate aminotransferase (AST), alanine aminotransferase (ALT), alkaline phosphatase, and gamma-glutamyl transpeptidase (GGT). Dialysis modality was defined as either the modality used at 90 days after dialysis initiation or, if death occurred before 90 days, the modality used at dialysis initiation. HBV infection was defined as positivity for HBsAg, and HCV infection as positivity for HCV Ab. At enrollment, HBsAg and HCV Ab titers were assessed using chemiluminescent microparticle immunoassay technology. Data were collected using a web-based platform (http://webdb.crc-esrd.or.kr). Date and cause of death were reported within one month after the event, and verified using data from Statistics Korea.

### Statistical Analysis

All data are expressed as median and range, or mean ± SD. Categorical data were compared using the chi-square test or Fisher’s exact test. Continuous data were compared using a t-test for differences between two groups, or one-way analysis of variance for differences among three groups. The linear-by-linear association test was used to analyze the association between hepatitis prevalence and dialysis vintage. To balance the baseline characteristics of HBV and HCV infected patients, we estimated the propensity score. The model was constructed to include age, sex, comorbid diabetes at baseline, creatinine, albumin, dialysis vintage, and dialysis modality. The Greedy match algorithm was used to create propensity score matched pairs without replacement. Survival rate during follow-up was analyzed using the Kaplan-Meier method. Cox proportional hazard models were used to calculate the hazard ratio (HR) and confidence interval (CI). A *p* value of <0.05 was considered significant.

## Results

Within the total study population of 3,336 patients, the prevalence of HBV infection was 7.1%, and the prevalence of HCV infection was 3.7%. Table 1 presents the characteristics of patients with hepatitis and patients without hepatitis virus infection in the unmatched cohort. Compared to patients without hepatitis infection, patients with HBV infection showed a lower age at dialysis initiation. There was a higher proportion of male patients within the hepatitis-infected group, compared to among those without hepatitis virus infection. With regard to comorbidities, lower proportions of patients showed diabetes, coronary artery disease, and chronic lung disease among those with HBV infection, compared to among patients without hepatitis virus infection. However, both HBV- and HCV-infected patients had a higher prevalence of moderate-to-severe chronic liver disease, compared to non-hepatitis-infected patients. Patients with HBV infection had higher levels of creatinine, AST, ALT and GGT than patients without hepatitis infection. Albumin levels were lower in patients with HCV infection than patients without hepatitis infection.

**Table 1:** Baseline characteristics of patients with hepatitis B, with hepatitis C, or without hepatitis infection.

	No hepatitis (n = 2992)	HBV (n = 221)	*p* value^a^	HCV (n = 108)	*p* value^a^
Age at dialysis initiation, mean ± SD	55.3 ± 14.3	50.3 ± 11.6	<0.001	55.0 ± 14.5	0.84
Type of dialysis, n (%)			0.07		0.70
HD	2239 (74.8)	153 (69.2)		79 (73.2)	
PD	753 (21.2)	68 (30.8)		29 (26.9)	
Male sex, n (%)	1701 (56.9)	157 (71.0)	0.00	74 (68.5)	0.02
Primary renal disease, n (%)			0.00		0.01
Diabetes	1424 (48.0)	93 (42.3)		60 (55.6)	
Hypertension	597 (20.1)	33 (15.1)		12 (11.1)	
Glomerulonephritis	421 (14.2)	55 (25.1)		9 (8.3)	
Others	523 (17.6)	38 (17.4)		27 (25.0)	
Comorbidity, n (%)					
Diabetes	1308 (51.3)	78 (42.9)	0.02	55 (59.1)	0.14
Congestive heart failure	260 (10.2)	13 (7.1)	0.15	7 (7.6)	0.42
Coronary artery disease	309 (12.2)	10 (5.5)	0.01	13 (14.1)	0.57
Peripheral vascular disease	140 (5.5)	4 (2.2)	0.05	9 (9.8)	0.08
Arrhythmia	51 (2.0)	4 (2.2)	0.91	1 (1.1)	0.54
Cerebrovascular disease	221 (8.7)	10 (5.5)	0.11	9 (9.8)	0.71
Chronic lung disease	178 (7.0)	5 (2.8)	0.02	10 (10.9)	0.16
Peptic ulcer disease	153 (6.0)	8 (4.4)	0.33	6 (6.5)	0.84
Moderate-to-severe liver disease	43 (1.7)	41 (22.5)	0.00	9 (9.9)	0.00
Malignancy	135 (5.3)	10 (5.5)	0.99	3 (3.3)	0.39
Laboratory data, mean ± SD					
Hemoglobin	9.8 ± 1.7	9.9 ± 1.8	0.68	9.8 ± 1.5	0.79
Blood urea nitrogen	70.2 ± 30.5	69.6 ± 29.0	0.79	69.3 ± 25.1	0.71
Creatinine	9.14 ± 3.50	9.80 ± 4.13	0.02	8.94 ± 2.82	0.48
Albumin	3.6 ± 0.6	3.6 ± 0.6	0.14	3.5 ± 0.6	0.03
Calcium	8.3 ± 1.1	8.3 ± 1.0	0.99	8.3 ± 0.9	0.95
Phosphorus	5.2 ± 1.7	5.2 ± 1.8	0.96	5.2 ± 1.5	0.83
Estimated GFR	6.44 ± 3.90	6.79 ± 6.86	0.46	6.49 ± 3.20	0.88
Urine volume	513.0 ± 584.8	441.5 ± 599.2	0.08	425.5 ± 515.4	0.13
AST	20.5 ± 25.0	27.4 ± 27.5	0.00	23.6 ± 21.5	0.15
ALT	18.3 ± 30.0	26.1 ± 27.2	0.00	22.3 ± 21.3	0.06
ALP	135.1 ± 135.5	137.9 ± 123.0	0.77	165.9 ± 265.5	0.24
GGT	33.7 ± 55.6	53.0 ± 87.9	0.02	37.8 ± 31.2	0.38

HBV, hepatitis B virus; HCV, hepatitis C virus; SD, standard deviation; HD, hemodialysis; PD, peritoneal dialysis; GFR, glomerular filtration rate; AST, aspartate aminotransferase; ALT, alanine aminotransferase; ALP, alkaline phosphatase; GGT, gamma-glutamyl transpeptidase.

^a^ versus no hepatitis.

**Fig 1:**
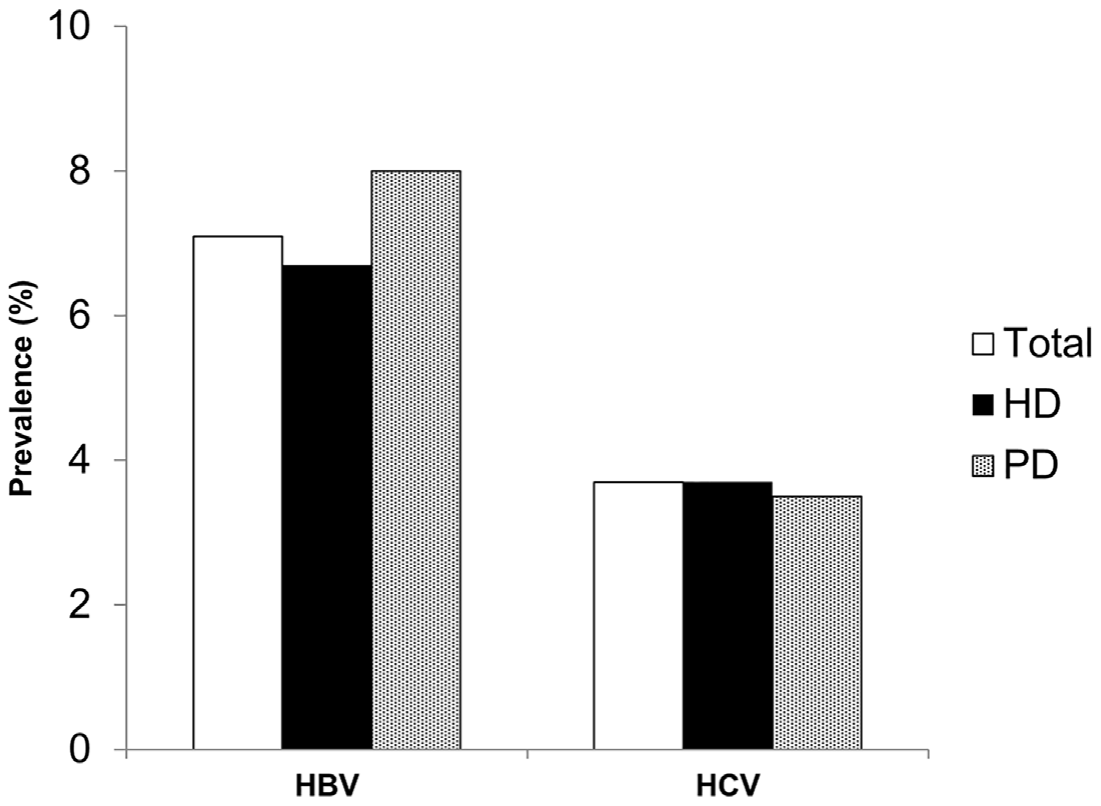
Prevalence of viral hepatitis infection according to dialysis modality. In our study population, we found a hepatitis B virus (HBV) infection prevalence of 7.1%, and a hepatitis C virus (HCV) infection prevalence of 3.7%. These prevalences did not significantly differ between dialysis modalities. HD, hemodialysis; PD, peritoneal dialysis.

**Fig 2:**
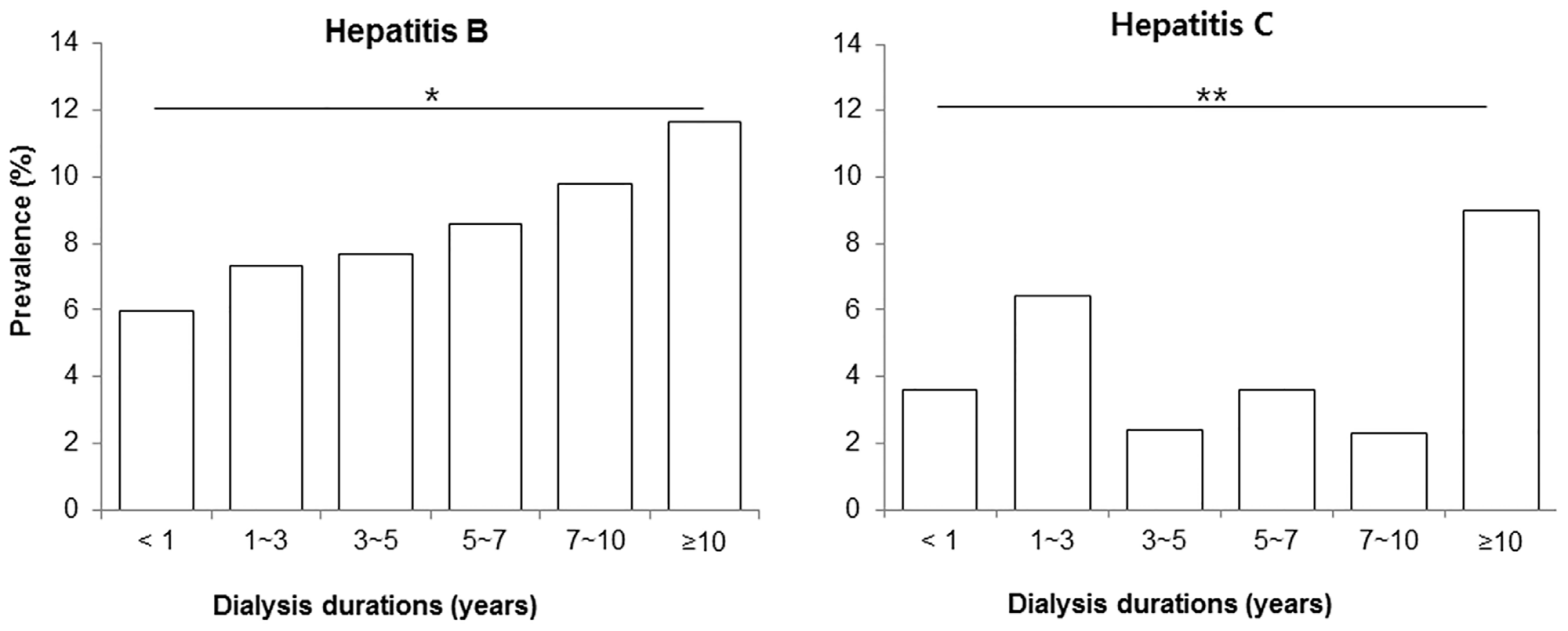
Prevalence of viral hepatitis infection according to dialysis vintage. Patients with dialysis vintage of ≥10 years showed significantly higher prevalences of HBV and HCV infection compared to patients with a dialysis vintage of <1 year. *p < 0.01, **p < 0.001 vs. group with dialysis vintage < 1 year.

The prevalences of HBV and HCV infection did not significantly differ according to dialysis modality (Fig 1). Fig 2 shows the prevalence of both HBV infection and HCV infection according to dialysis vintage. Linear-by-linear association analysis revealed a significantly higher prevalence of HBV infection associated with increasing dialysis vintage (*p* = 0.001). Similarly, HCV infection prevalence tended to rise with increasing dialysis vintage (*p* = 0.074). Specifically, the prevalences of HBV and HCV infection were significantly higher among patients with dialysis vintage longer than 10 years, compared to patients with dialysis vintage of less than 1 year (*p* = 0.003 for HBV and *p* < 0.001 for HCV).

Baseline demographics of patients with HBV or HCV infection were compared with those of the matched cohort (Table 2), with propensity score matching used to adjust baseline confounding variables to mortality. After propensity score matching, patients with and without hepatitis did not significantly differ in any of the variables used in scoring—except coronary artery disease, which remained more prevalent among patients with HBV infection (*p* = 0.04). AST, ALT, and GGT were significantly higher in the HBV infection group. None of our patients with HCV infection were diagnosed with cryoglobulinemia. Moderate-to-severe liver disease had a higher prevalence among patients with HBV or HCV infection compared to patients without hepatitis virus infection. Of the patients in our present study with HBV infection, 15 were treated with antiviral agents, including entecavir in 8 patients, lamivudine in 5 patients, and adefovir in 2 patients. None of the HCV infected patients were treated with any antiviral agents.

**Table 2:** Baseline characteristics of propensity score-matched hepatitis B and C populations.

	Hepatitis B			Hepatitis C		
	No hepatitis (n = 358)	HBs Ag-positive (n = 179)	*p* value^a^	No hepatitis (n = 180)	HCV Ab-positive (n = 90)	*P* value^a^
Age at dialysis initiation, mean ± SD	49.7 ± 14.9	50.3 ± 11.8	0.59	55.2 ± 13.7	55.9 ± 13.9	0.69
Type of dialysis, n (%)			0.80			0.32
HD	236 (65.9)	120 (67.0)		138 (76.7)	64 (71.1)	
PD	122 (34.1)	59 (33.0)		42 (23.3)	26 (28.9)	
Male sex, n (%)	249 (69.6)	123 (68.7)	0.84	119 (66.1)	63 (70.0)	0.52
Comorbidity, n (%)						
Diabetes	144 (40.2)	78 (43.6)	0.46	105 (58.3)	55 (61.1)	0.66
Congestive heart failure	34 (9.5)	12 (6.7)	0.27	17 (9.4)	7 (7.9)	0.67
Coronary artery disease	39 (11)	10 (5.6)	0.04	30 (16.7)	13 (14.6)	0.66
Peripheral vascular disease	18 (5.0)	4 (2.3)	0.13	12 (6.7)	9 (10.1)	0.32
Arrhythmia	7 (2.0)	4 (2.2)	0.83	4 (2.2)	1 (1.1)	0.53
Cerebrovascular disease	23 (6.4)	10 (5.6)	0.70	12 (6.7)	9 (10.1)	0.32
Chronic lung disease	24 (6.7)	5 (2.8)	0.06	12 (6.7)	10 (11.2)	0.20
Peptic ulcer disease	25 (7.0)	8 (4.5)	0.25	11 (6.1)	6 (6.7)	0.84
Moderate-to-severe liver disease	6 (1.7)	41 (22.9)	0.00	4 (2.2)	9 (10.2)	0.00
Malignancy	17 (4.8)	10 (5.6)	0.67	11 (6.1)	3 (3.4)	0.35
Laboratory data, mean ± SD						
Hemoglobin	9.6 ± 1.8	9.6 ± 1.8	0.85	9.8 ± 1.6	9.7 ± 1.5	0.50
Blood urea nitrogen	73.5 ± 34.1	71.8 ± 30.6	0.57	64.8 ± 24.1	70.5 ± 26.8	0.08
Creatinine	9.86 ± 3.73	9.80 ± 4.30	0.88	8.78 ± 3.34	8.87 ± 2.89	0.82
Albumin	3.54 ± 0.61	3.49 ± 0.64	0.38	3.48 ± 0.64	3.47 ± 0.65	0.87
Calcium	8.1 ± 1.2	8.2 ± 1.1	0.48	8.2 ± 1.0	8.2 ± 1.0	0.75
Phosphorus	5.3 ± 1.9	5.3 ± 2.0	0.87	5.1 ± 1.4	5.2 ± 1.5	0.58
AST	20.6 ± 15.3	27.2 ± 28.0	0.00	25.3 ± 72.3	24.4 ± 23.0	0.91
ALT	17.3 ± 14.9	24.7 ± 26.2	0.00	21.6 ± 64.36	23.15 ± 22.40	0.83
ALP	132.1 ± 127.6	136.0 ± 125.5	0.74	133.9 ± 146.9	140.8 ± 137.1	0.72
GGT	34.0 ± 59.2	53.0 ± 89.2	0.04	30.25 ± 32.9	36.83 ± 30.4	0.24

SD, standard deviation; HD, hemodialysis; PD, peritoneal dialysis; AST, aspartate aminotransferase; ALT, alanine aminotransferase; ALP, alkaline phosphatase; GGT, gamma-glutamyl transpeptidase.

^a^ Comparisons were made using chi-square, Fisher’s exact test, or t-test as appropriate.

During the mean follow-up of 23.5 ± 13.3 months in the matched cohort, 28 HBsAg-positive patients died, with infection being the most common cause of death (35.7%). Among the HCV Ab-positive patients, there were 22 deaths, and cardiovascular disease was the most common cause of death in this group (40.9%). Causes of death in the control group did not significantly differ from those in either HBsAg-positive or HCV Ab-positive patients (Table 3).

**Table 3:** Causes of death within the matched hepatitis B and C populations.

Cause of death	Hepatitis B		Hepatitis C	
	No hepatitis (n = 41)	HBs Ag-positive (n = 28)	No hepatitis (n = 24)	HCV Ab-positive (n = 22)
Cardiovascular disease, n (%)	17 (41.5)	8 (28.6)	8 (33.3)	9 (40.9)
Infection, n (%)	13 (31.7)	10 (35.7)	6 (25.0)	5 (22.7)
Liver disease, n (%)	1 (2.4)	2 (7.1)	0 (0.0)	0 (0.0)
Malignancy, n (%)	2 (4.9)	2 (7.1)	1 (4.2)	1 (4.6)
Others, n (%)	4 (9.8)	2 (7.1)	4 (16.7)	2 (9.1)
Unknown, n (%)	4 (9.8)	4 (14.3)	5 (20.8)	5 (22.7)
Total, n	41	28	24	22

HBsAg, hepatitis B surface antigen; HCV Ab, hepatitis C virus antibody.

**Fig 3:**
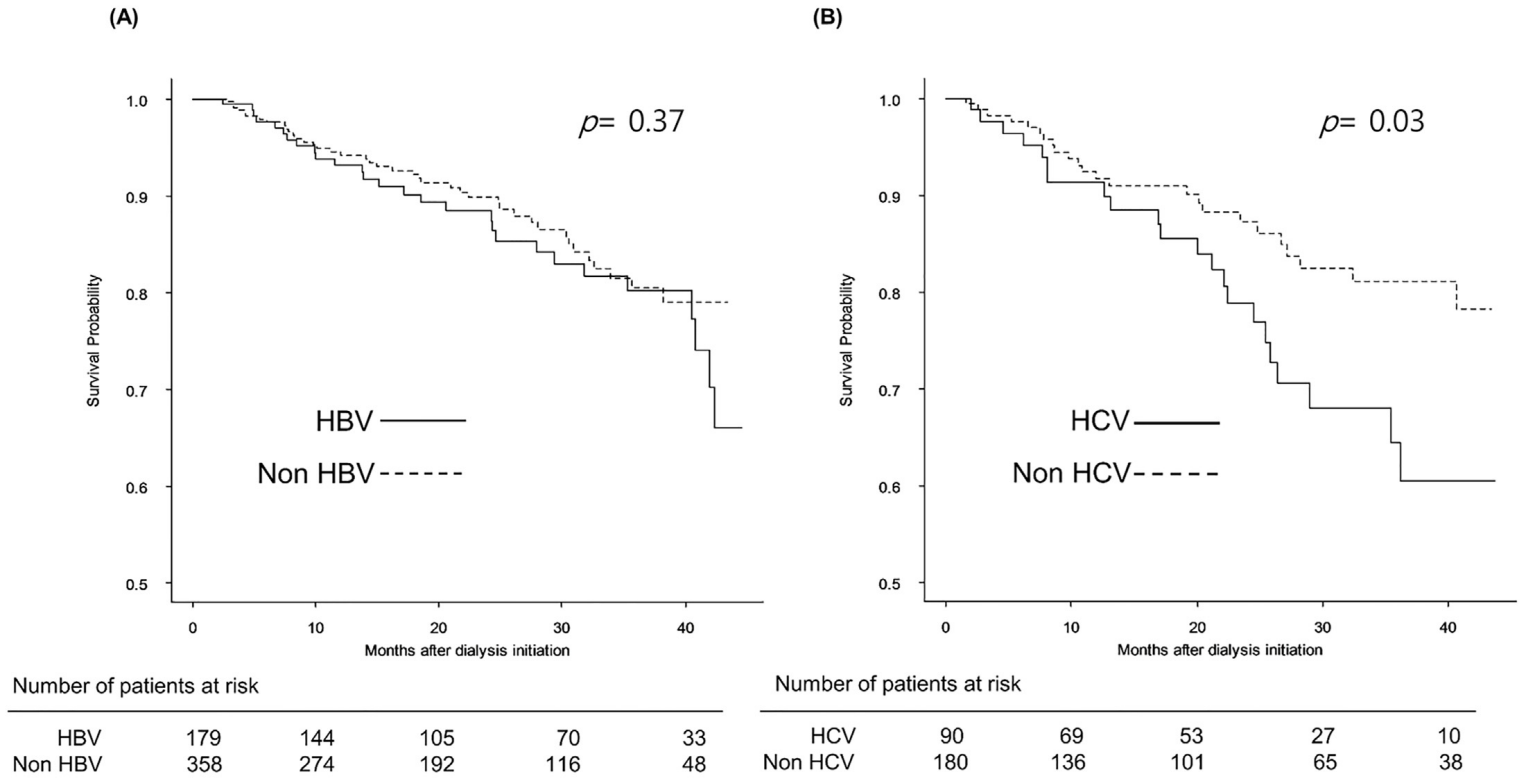
Kaplan—Meier survival curves of the propensity score-matched cohort. (A) Survival probability did not significantly differ based on HBV infection (*p* = 0.37). (B) HCV infection was associated with a lower survival probability (*p* = 0.03).

Fig 3 shows Kaplan-Meier plots for survival in the matched cohort for the HBsAg-positive and HCV Ab-positive groups. Cumulative survival at 36 months was 80.2% in the HBsAg-positive group and 80.4% in the HBsAg-negative group, showing no significant different between groups (*p* = 0.37). In contrast, cumulative 36-month survival rates in HCV Ab-positive patients (64.5%) were significantly lower than among HCV Ab-negative controls (81.0%) (*p* = 0.03).

**Fig 4:**
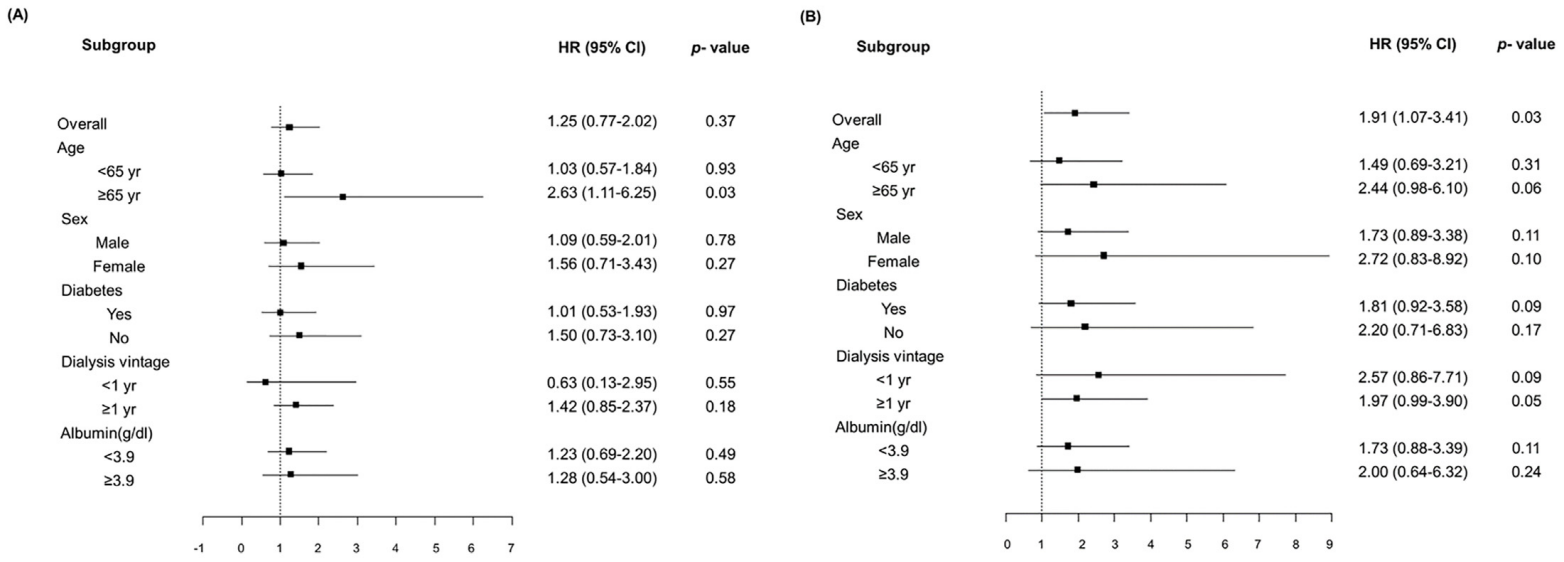
Subgroup analysis of HBV and HCV infection. (A) HBV infection was more strongly associated with mortality among patients of ≥65 years of age (HR, 2.63; 95% CI, 1.11–6.25; *p* = 0.03). (B) HCV infection showed no specific association with mortality in any of the subgroups.

The crude HR of all-cause mortality was 1.25 (95% CI, 0.77–2.02; *p* = 0.37) for HBV infected patients and 1.91 (95% CI, 1.07–3.41; *p* = 0.03) for HCV infected patients. The association between HBV infection and all-cause mortality did not significantly differ with regards to diabetes, sex, dialysis vintage, or albumin level. HBV infection was more strongly associated with death among patients of ≥65 years of age (HR, 2.63; 95% CI, 1.11–6.25; *p* = 0.03) (Fig 4).

## Discussion

In the present results, HBV infection was positively associated with dialysis vintage, and both HBV and HCV infection prevalences were highest among patients with the longest dialysis vintage. On the other hand, dialysis modality did not influence the prevalence of viral hepatitis infection. HBV infection did not affect survival rate, however, HCV infection was associated with an increased risk of mortality among maintenance dialysis patients. To the best of our knowledge, this is the first study to simultaneously compare the impacts of both HBV and HCV infections on survival among dialysis patients.

Previous studies have reported that HBs Ag and HCV Ab positivity are related to longer dialysis duration [15, 16]. This is consistent with our present finding that patients with the longest dialysis vintage showed significantly higher HBV and HCV infection prevalences compared to patients with the shortest dialysis vintage. This finding is likely explained by the association between hepatitis infection risk and increased exposure to blood, blood products, and contaminated equipment in the areas surrounding where dialysis is performed [17, 18]. In Korea, separate dialysis machines are used for HBsAg-positive patients, but such preventive measures are not applied for HCV Ab-positive patients.

The influence of dialysis modality on hepatitis infection remains controversial. Several studies have identified hemodialysis as a major risk factor for viral hepatitis infection [19–23]. However, substantial variability has been observed across Asian-Pacific areas, with no difference in annual incidence observed between patients receiving hemodialysis and peritoneal dialysis. Viral hepatitis prevalence could conceivably be influenced by the frequency in the underlying population rather than by dialysis modality, especially in areas of viral hepatitis infection endemicity, including the Korean population in our present study [24, 25]. Future studies should examine the prevalence of viral hepatitis in different countries with serial follow-up, giving consideration to the variable seroprevalence within different populations and different clinical situations.

In our present study, clinical outcomes of ESRD patients were analyzed using the propensity score matching method to control confounding factors. The survival curve of the propensity score-matched cohort showed that HBV infection did not significantly impact survival. Few prior studies have reported the effect of HBV infection on survival among patients receiving maintenance dialysis. Josselson et al. investigated 30 HBsAg-positive and 64 HBsAg-negative patients undergoing dialysis, and observed no differences in survival, causes of death, or hospitalization between the two groups [13]. Harnett et al. reported similar findings in a study including 49 hemodialysis patients with HBV infection [26]. In contrast, Jha et al. investigated 11 HBsAg-positive dialysis patients, and showed that seropositivity was associated with higher rates of death and liver failure, compared with those rates in HBsAg-negative patients [14]. However, these studies were limited by small sample sizes, insufficient statistical power, and retrospective design, and were conducted before the introduction of nucleoside antiviral agents. Our present study included a larger number of patients with HBV infection, and demonstrated that HBV-infected dialysis patients showed survival comparable to that of patients without HBV infection. Subgroup analysis revealed that HBV infection was associated with significantly higher mortality among dialysis patients older than 65 years. The pathogenic mechanism by which HBV infection specifically influences mortality in older age is unclear. However, this finding may suggest a long-term effect of HBV infection on mortality, warranting an extended follow-up study of HBV-infected patients.

Treatment of viral hepatitis infection should be considered in survival analyses of patients with HBV infection. Nucleoside drugs represent an opportunity to improve the natural history of HBV infection. Lamivudine treatment reportedly leads to a high overall rate of HBV DNA clearance, even in dialysis patients [27, 28], and several retrospective studies have shown beneficial effects of lamivudine treatment [29–34]. However, no controlled trials have investigated the effects of antiviral treatment in HBV-infected patients receiving dialysis. Our present data demonstrated comparable survival between dialysis patients with and without HBV infection, but only scarce information was available relating to the prescription of antiviral agents. Further studies are needed to investigate whether favorable outcomes in cases of HBV infection are related to the clinical feature of being a chronic carrier [16, 24] or to the effects of antiviral treatment.

In contrast to HBV infection, HCV infection was associated with significantly lower survival compared with patients without HCV infection in our present study. Most studies in the United States and Europe have reported that HCV Ab-positive patients receiving maintenance dialysis exhibit an increased risk of all-cause mortality [6–8, 35, 36]. However, only sparse data are available comparing the mortality of dialysis patients with and without HCV infection in Asian countries [37, 38]. Recently, a Japanese study also concluded that HCV Ab-positivity was independently associated with higher mortality after adjusting for traditional risk factors [39]. Overall, it appears likely that HCV infection increases the risk of all-cause mortality among Asian dialysis patients.

Pegylated interferon (IFN) and ribavirin combination therapy is the standard HCV treatment in the general population [40]. However, limited information is available regarding the use of this combined therapy for HCV infection among patients on dialysis. Ribavirin is contraindicated in patients with creatinine clearance of less than 50 mL/min, since it is not removed by dialysis and accumulated doses can cause severe hemolytic anemia [41]. The Kidney Disease Improving Global Outcomes (KDIGO) clinical guidelines suggest the use of monotherapy with IFN for patients receiving hemodialysis [42]. The fact that HCV-infected dialysis patients cannot receive optimal treatment might explain their lower survival compared with HBV-infected dialysis patients. Further research is needed to investigate options for treating viral hepatitis infection in patients receiving maintenance dialysis.

The present liver function test results showed significantly higher levels among HBsAg-positive patients compared with patients without hepatitis infection. Although still within normal ranges, the increased AST, ALT, and GGT levels in HBsAg-positive patients persisted after propensity score matching. This suggests that although hepatitis infection appears asymptomatic and indolent, it is independently associated with hepatocellular injury [43, 44]. The indicated hepatocellular injury is most likely attributable to the chronic inflammation and autoimmune mechanisms derived from viral hepatitis infection [45, 46]. Moreover, HCV infection may intensify oxidative stress among patients with uremia, which is associated with cardiovascular mortality [47]. This underlying inflammatory state may be associated with the higher death risk among patients with HCV infection and the higher proportion of infection-related deaths among patients with HBV infection.

Our study has several limitations. First, since serologic status was confirmed at the time of study enrollment, we do not know when the patients were infected with HBV or HCV. However, all-cause mortality was increased both in patients who were HCV Ab-positive at baseline, and those who developed HCV Ab-positivity during the course of dialysis [5]. This suggests similar impacts of prevalent and incident infections with viral hepatitis among dialysis patients. Second, anti-HCV enzyme immunoassay tests have a high false-negative rate among patients on maintenance dialysis, especially for patients undergoing a longer duration of dialysis [48]. More sensitive diagnostic tests, such as HCV RNA, could detect false-negative HCV Ab patients [49]. However, as this was a multicenter observational cohort study, no further information was collected relating to HCV RNA tests. Moreover, enzyme immunoassay tests are the most commonly used method for evaluating HCV infection among dialysis patients, and most previous studies have reported consistent outcomes using this method. Finally, the natural history of HBV infection and HCV infection infections extend over decades; thus, their adverse consequences may not be obvious during our relatively short follow-up period [27]. Nevertheless, our study is the first nationwide prospective study to use a propensity matched cohort to compare the outcomes of these two viral hepatitis infections among dialysis patients.

In conclusion, here we identified significant increases in the prevalences of HBV and HCV among patients with the longest dialysis vintage. While HBV infection was not associated with significantly altered mortality, HCV infection increased the risk of mortality among patients receiving maintenance dialysis. Although within the normal reference range, hepatitis patients tended to have higher levels of liver function test variables. Hepatitis infection may result in a state of asymptomatic hepatocellular injury with ongoing inflammation. Further studies are needed to evaluate the effects of underlying inflammation associated with hepatitis infection, and the long-term natural history of hepatitis infection among dialysis patients.
